# Integrated femtosecond-laser workflow for decapsulation, delayering, and cross-sectioning of advanced package-on-package devices

**DOI:** 10.1038/s41598-026-56063-x

**Published:** 2026-06-03

**Authors:** Mohammad Taghi Mohammadi Anaei, Matthew Maniscalco, Hongbin Choi, Wesley Roser, Marcus Emanuel, Adrian Phoulady, Parisa Mahyari, Alexander Blagojevic, Nicholas May, Garth C. Egan, Sina Shahbazmohamadi, Pouya Tavousi

**Affiliations:** 1 University of Connecticut, Storrs, CT USA; 2 The MITRE Corporation, Bedford, MA USA; 3 Tescan Group, Storrs, CT USA

**Keywords:** Engineering, Materials science, Optics and photonics

## Abstract

Advanced packaging devices integrate multiple heterogeneous materials and components (e.g., mold compounds, redistribution/interposer structures, stacked memory, and logic dies) into compact assemblies whose failures can propagate into high-impact system-level outages. High-resolution, layer-by-layer 3D imaging is therefore essential for (i) failure analysis (FA) and root-cause determination, (ii) verification and validation (V&V) via as-designed versus as-built comparison, and (iii) security-focused inspection aimed at identifying unintended or malicious structural modifications. However, conventional approaches—mechanical polishing, chemical etching, and focused ion beam (FIB) delayering—are often slow, limited in precision, inconsistent over large areas, or difficult to scale for systematic workflows. In this work, we have shown that laser delayering can promise an effective alternative that is simultaneously fast and precise. Here, we demonstrate an integrated femtosecond-laser workflow for advanced package-on-package deprocessing that combines decapsulation, DRAM/component removal, sequential redistribution-layer exposure and removal, and targeted logic-die cross-sectioning on a single laser-processing platform. Process outcomes were validated using laser-scanning confocal microscopy for high-fidelity surface topography and scanning electron microscopy (SEM) for microstructural assessment. The results demonstrate a practical, rapid, and reproducible pathway for advanced-package system deconstruction and design reconstruction workflows supporting FA, V&V, and hardware assurance.

## Introduction

The ability to reliably reveal and reconstruct internal features in modern semiconductor hardware is foundational to failure diagnostics, process optimization, and design validation in advanced electronics^1–4^. This need is amplified in advanced packaging^5,6^, where tightly integrated multi-material stacks increase both the likelihood and the consequence of latent defects. When failures occur in such devices, the downstream impact can extend from degraded performance and yield loss to catastrophic failure in safety- or mission-critical systems. Rapid and repeatable 3D imaging workflows enable root-cause failure analysis (FA) by linking observed damage mechanisms and anomalies to specific process conditions and structural outcomes. In parallel, verification and validation (V&V) benefits from direct comparison of as-designed versus as-built structures, supporting identification of design-rule violations, manufacturing excursions, and hidden defects that may otherwise remain undetected until field failure^3,4^. For critical systems, this same capability supports hardware assurance by enabling inspection for unintended structural modifications, including potential hardware Trojans or malicious alterations introduced during fabrication or assembly^7^.

Despite this importance, today’s mainstream layer-exposure approaches face growing limitations as architectures become more complex and dimensions shrink. Mechanical polishing and chemical etching are inherently prone to variability and can induce damage, incomplete removal, or loss of critical interfaces, particularly across heterogeneous material stacks. Focused ion beam (FIB) delayering offers excellent precision and is widely used in FA, but its low material removal rate makes it impractical for large-area delayering, iterative workflows, or high-throughput environments^8–11^. Non-destructive imaging methods such as laboratory X-ray CT typically lack the resolution to resolve fine interconnects and sub-micron features, while synchrotron-based nano-CT can achieve the needed resolution but is costly and inaccessible for routine use^12,13^.

Laser-based delayering has emerged as a compelling alternative because it is non-contact, fast, and potentially scalable. Ultrafast pulsed lasers, in particular, can ablate with minimal thermal diffusion, helping preserve underlying structures and interfaces. In our earlier studies using IR ultrashort-pulsed lasers, we demonstrated rapid delayering capability but observed some limitations. In our recently investigated green (515 nm) ultrashort-pulsed laser delayering^14–16^, we achieved significant improvements. Specifically, through systematic parameter exploration, we achieved cleaner interfaces, reduced debris, and improved visibility of interconnect and dielectric features—validated via confocal microscopy, and SEM—on bare dies. However, advanced packaging introduces additional layers, larger thickness variation, and broader material diversity, creating a strong need to translate and validate these methods on real packaged devices^8,9,17–20^.

In this work, we demonstrate an all-in-one femtosecond-laser workflow for an advanced package-on-package device, starting from decapsulation and proceeding through sequential delayering of package-level layers, interposer/redistribution structures, DRAM components, and the logic die, complemented by targeted cross-sectioning to reveal internal layer arrangement. Using multimodality microscopy (confocal and SEM), we showcase a practical route toward reproducible system deconstruction and design reconstruction workflows for FA, V&V, and hardware assurance in advanced packaged semiconductors.

To clarify the specific advance of the present study relative to our prior work and recent literature, Table 1 compares the scope of the present workflow with earlier femtosecond-laser delayering, laser/FIB hybrid preparation, and green-laser bare-die studies. Previous work from our group demonstrated femtosecond-laser delayering, volumetric reconstruction, laser/FIB hybrid preparation, and high-fidelity laser delayering primarily on PCBs, bare dies, or selected IC regions. In contrast, the present work extends these methods to a real advanced package-on-package device, where the workflow begins with epoxy molding-compound decapsulation and proceeds through DRAM removal, exposure and removal of interposer/redistribution-layer Cu structures, and targeted logic-die cross-sectioning. Thus, the main advance is not only the use of a femtosecond laser for material removal, but the integration of multiple package-analysis operations on a single platform and on a single advanced packaged device.

**Table 1:** The scope of the present workflow versus earlier femtosecond-laser delayering, laser/FIB hybrid preparation, and green-laser bare-die studies.

Work / literature category	Sample type	Demonstrated capability	Limitation relative to present work	Advance in present work
Prior laser-delayering and volumetric imaging work	PCB or selected electronic structures	Rapid laser delayering and 3D reconstruction	Did not demonstrate advanced package-on-package deprocessing	Extends workflow to heterogeneous advanced package
Prior chip reverse-engineering work	Bare die / selected IC regions	Laser delayering, laser/FIB hybrid polishing, volumetric imaging	Focused on chip-level/bare-die analysis, not package-level decapsulation and DRAM/RDL removal	Adds full package-level access before die inspection
Prior 515 nm green-laser delayering work	Bare semiconductor die	Improved layer clarity, reduced debris, better feature visibility compared with IR	Did not include molding compound, DRAM stacks, interposer, or package RDL structures	Applies green femtosecond processing to a broader heterogeneous package stack
FIB / PFIB approaches	Local die/package regions	High precision, high-resolution local milling	Low throughput for large-area package deprocessing	Uses femtosecond laser for rapid access over larger areas
Present work	Advanced package-on-package device with TSMC 5 nm SoC	Decapsulation, DRAM removal, Cu RDL delayering, targeted logic-die cross-sectioning	Full logic-die layer-by-layer reconstruction remains future work	Demonstrates an integrated single-platform package deprocessing workflow

## Materials and methods

### Sample

**Fig. 1:**
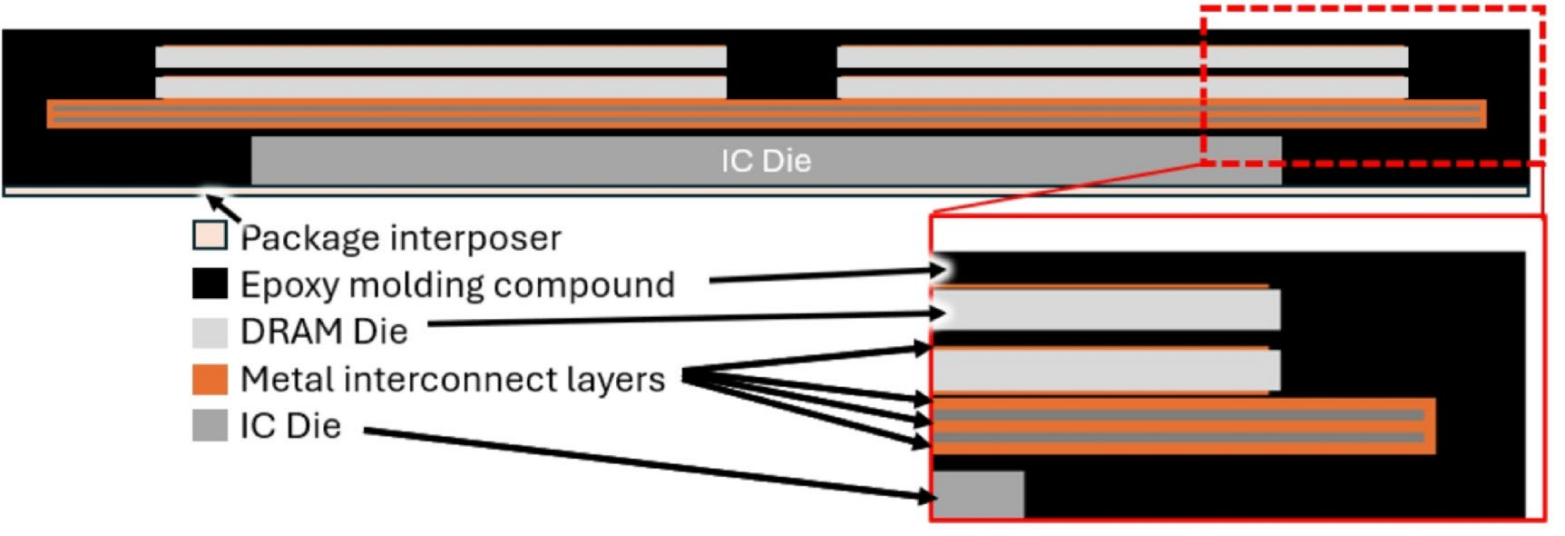
Schematic of the package-on-package device under study.

This study focuses on an advanced package-on-package device with a TSMC 5 nm process system-on-a-chip IC. The configuration of the package is shown in the cross-sectional schematic in Fig. 1. It is made up of the system-on-a-chip IC die mounted on an interposer. This side will be referred to as the bottom of the package. On top of the die is three-layer interposer with two stacks of two DRAM die. All this is secured in potting or molding epoxy-based material. Prior to processing, this package was removed from the underlying board.

### Femtosecond laser processing system

Laser processing was performed using a TESCAN FemtoChisel platform equipped with an ultrashort-pulsed femtosecond laser source capable of operation at 515 nm (green) and 1030 nm (IR), with 515 nm being used in this study. The source provides sub-300 fs pulse widths (fundamental) and supports repetition rates from single-shot operation up to the MHz range, with maximum average output power of 20 W. The beam was routed through a SCANLAB intelliSCANse 20 galvanometer scanner and focused onto the sample using telecentric F-Theta optics (70 mm focal length for IR operation and 75 mm focal length for green operation).

The delayering workflow for the advanced package proceeded from (i) femtosecond-laser-enabled decapsulation to access the package interior, followed by (ii) sequential femtosecond-laser delayering across package/interposer regions and memory structures, and (iii) preliminary exploration of laser delayering on the logic die. After each round of laser processing, CO_2_ cleaning was used to remove accumulated debris^17^.

### Parameter optimization strategy

To identify operating conditions that balance removal quality with throughput, key process variables governing ablation behavior and resulting surface quality were organized and evaluated, including (but not limited to) average power (or pulse energy), repetition rate, scan speed, number of passes/cycles, pulse-format settings (e.g., burst mode where applicable), and pulse width. A one-variable-at-a-time strategy was applied initially by holding parameters constant while varying a single factor to isolate its effect on delayering quality. Because multiple parameter combinations can yield comparable surface outcomes, final selection emphasized both (i) consistent exposure of underlying structures with minimal debris and (ii) reduced runtime to support practical workflows.

The 515 nm green wavelength was selected because it offers several advantages over 1030 nm IR processing for heterogeneous semiconductor packages. First, the shorter wavelength improves focusability, enabling a smaller effective spot size and more spatially confined material interaction. Second, based on our prior comparison of IR and green femtosecond delayering, the green wavelength reduces sensitivity to material-dependent absorption differences and produces cleaner layer exposure, reduced debris, and improved visibility of metal/dielectric features. These advantages are particularly important in advanced packages, where epoxy molding compound, silica filler, silicon-rich memory regions, dielectrics, and Cu redistribution layers must be processed sequentially.

Burst-mode pulse shaping was used selectively to improve removal quality in materials where single-pulse processing produced nonuniform ablation or excessive residual debris. For the epoxy molding compound, a use of burst mode improved removal uniformity compared with single-pulse operation under otherwise similar conditions. For DRAM removal, a shorter pulse spacing was found to perform better. In contrast, Cu-rich redistribution layers did not require high-burst operation; instead, longer pulse-width conditions were more effective for metal removal. Therefore, the workflow uses material-specific pulse formatting rather than a single universal laser condition.

### Post-processing imaging and characterization

#### Confocal microscopy

Post-delayering 3D surface imaging was performed using a Keyence VK-X3100 laser-scanning confocal microscope. Objective lenses of 20X, 50X, and 150X magnification were used. Images were acquired at 1024 × 768 pixels. Based on calibrated fields of view, effective pixel sizes were 1.37 × 1.37 μm/pixel (20X), 0.27 × 0.27 μm/pixel (50X), and 0.094 × 0.094 μm/pixel (150X). These settings enabled high-fidelity visualization of topography and fine structural features necessary for layer identification and reconstruction.

### SEM

High-resolution imaging was performed using a ZEISS Crossbeam 340 and a Thermo Fisher Scientific TENEO LV SEM. Imaging was conducted under high-vacuum conditions using secondary electron (SE) and backscattered electron (BSE) detectors to capture topographic and compositional contrast. Accelerating voltages were selected between 5 and 20 kV depending on the feature/material of interest, with working distances typically maintained between 5 and 10 mm. Magnifications ranged from ~ 500X to > 100,000X as required. To mitigate charging on non-conductive or partially delayered regions, a thin conductive sputter coat (e.g., Au or Pt) was applied when necessary prior to SEM analysis.

## Results

### Epoxy molding layer removal (Decapsulation)

As discussed in the Methods and Materials section, the advanced package-on-package device features a transposer/interposer-facing side referred to as the bottom of the sample. The top side is fully covered by a black epoxy molding compound. In this work, we begin the workflow from the top by removing the epoxy layer entirely to expose underlying package features for subsequent delayering and imaging.

**Fig. 2:**
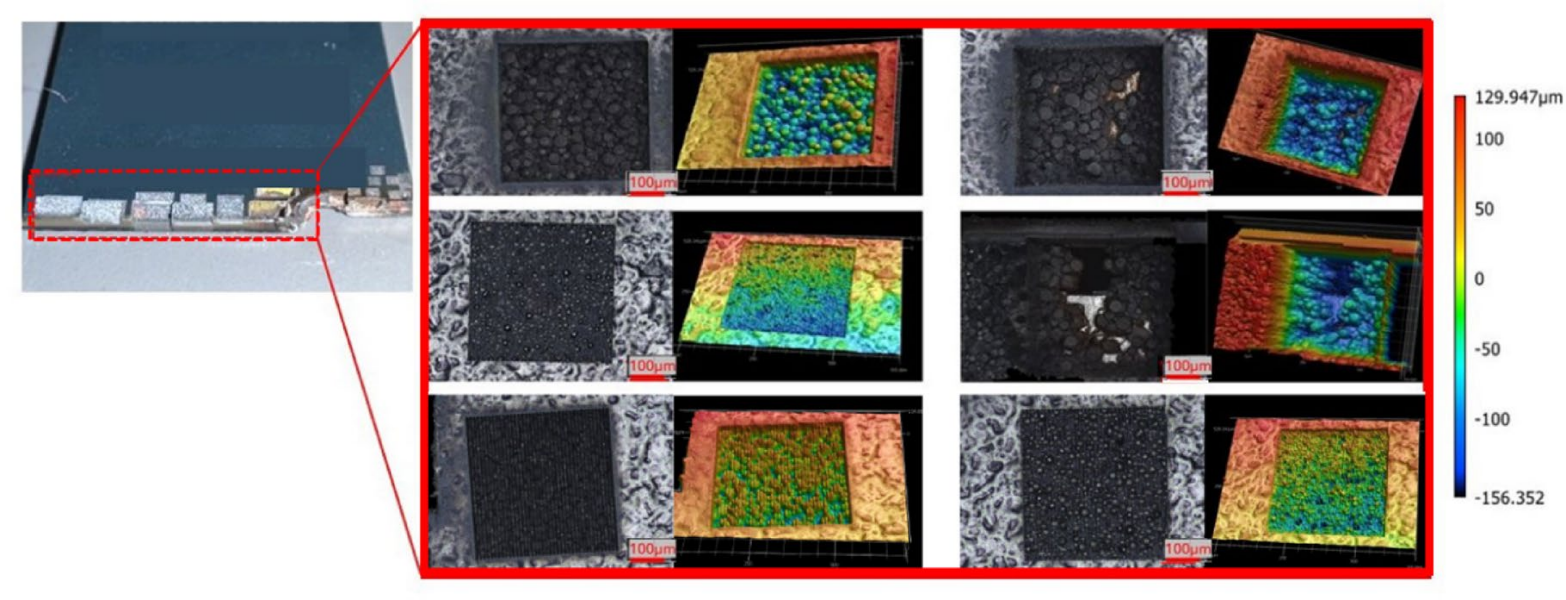
Non-optimized laser decapsulation parameters lead to failure. Some instances are demonstrated. Identifying information on device has been obscured for sensitivity reasons.

Laser-based removal of this material is non-trivial because the molding compound contains silica microsphere filler, which exhibits a significantly different response to the laser in comparison to the epoxy matrix. With non-optimized laser parameters, this leads to formation of irregular and porous surfaces (Fig. 2). As ablation proceeds, height variances in the resulting surfaces increase and the local interaction becomes less predictable, increasing the risk of damaging the underlying layer before the molding is fully cleared.

**Fig. 3:**
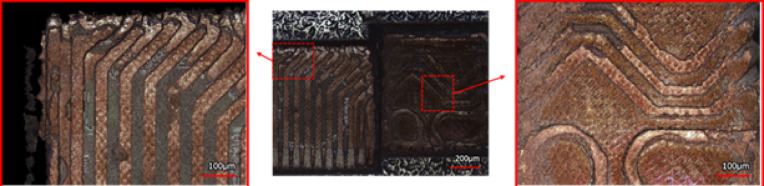
Burst mode effects on ablation rate: Left: no burst; Right: 9-ps burst. All other parameters were the same. Lower images show confocal depth maps of the same regions.

Based on prior findings in the literature^21–23^ and preliminary testing, we observed that molding removal is significantly improved using high-power operation combined with burst-enabled pulse formatting, which sends 2 or more pulses separated by either picoseconds or nanoseconds with each repetition rate cycle instead of the single pulse. This was found to enhance removal efficiency in polymeric and other non-metallic materials while promoting cleaner exposure of the underlying surface. An example comparing the approaches is shown in Fig. 3 where it can be seen that non-metal materials were removed more uniformly and with substantially reduced residual debris, enabling clean exposure of the underlying structure with a burst of 2 pulses 9 ps apart compared to the single pulse (no burst).

**Fig. 4:**
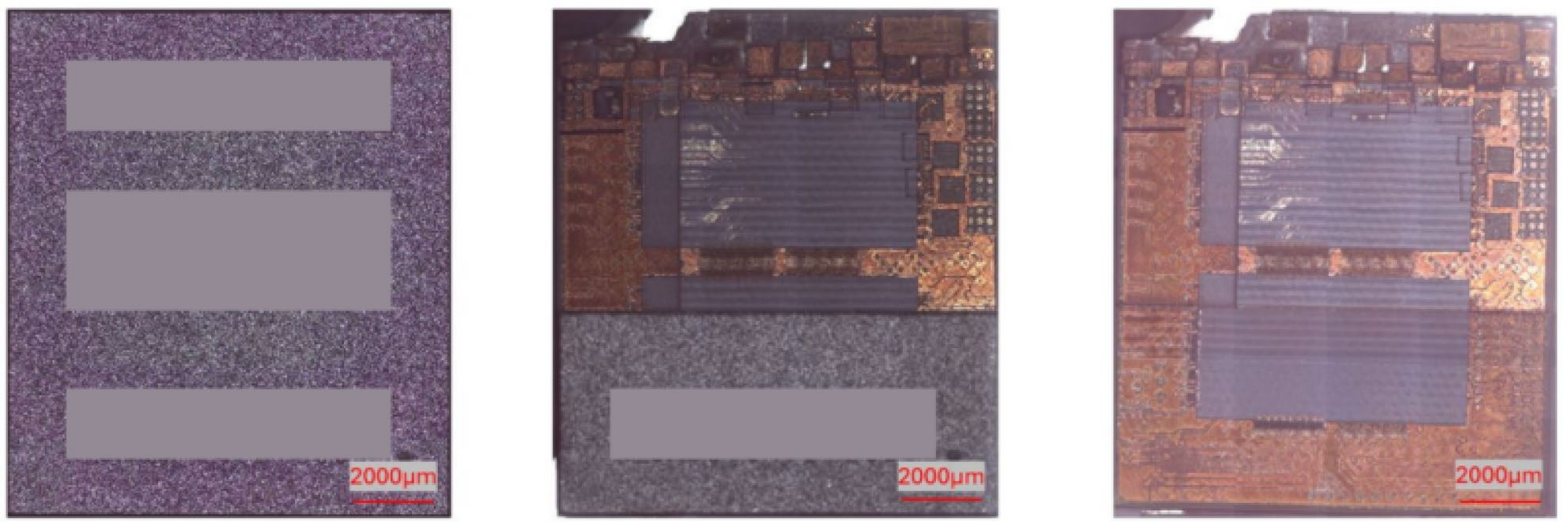
Left: Intact device. Middle: Partial decapsulation of the upper half. Right: Full-device decapsulation. Identifying information on devices has been obscured for sensitivity reasons.

After determining the optimal parameters, we applied them to the entire top of the package, resulting in complete epoxy removal (Fig. 4). The smaller square-shaped regions in the top right of the decapsulated views indicate parameter-optimization test areas used during process development, prior to the laser processing of the entire package area. Notably, all process development and the complete delayering workflow were performed on a single package, highlighting the robustness and practical reliability of the proposed method.

**Fig. 5:**
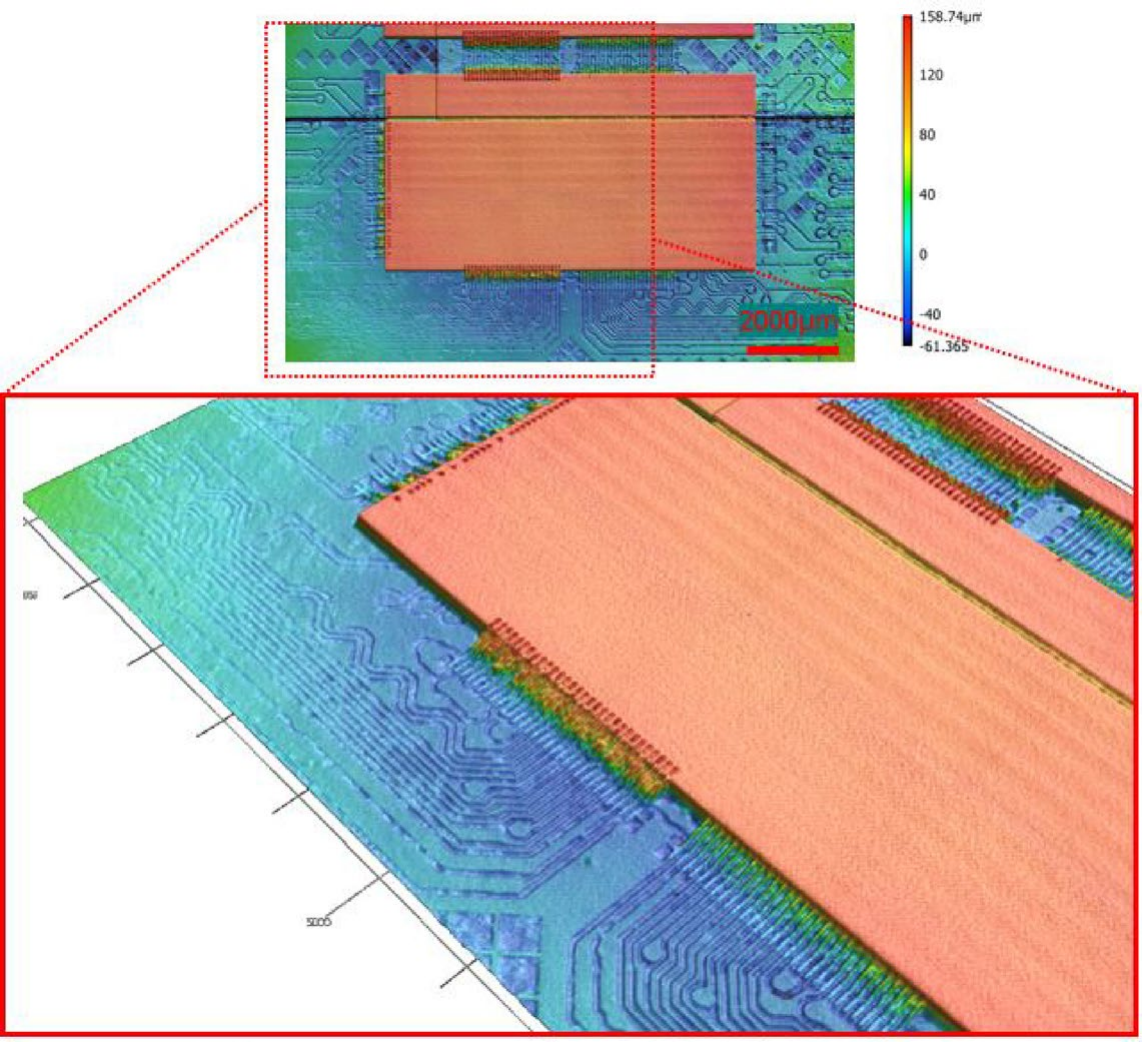
Confocal investigation of the decapsulated device highlights the cleanliness of the decapsulation procedure. Note the line through the DRAM towards the top of the field of view resulted from an overlapping of the two decapsulation cycles. Quantitative surface measurements are provided in Surface Topography Measurement Section.

Figure 5 shows a confocal measurement of the surface of decapsulated device, highlighting the cleanliness of the decapsulation procedure. Further, Table 2 provides SEM images from different regions of the decapsulated device as a way to assess the quality of the surfaces resulting from laser processing. The images show that the polymer-based materials in both the molding compound and top layer of the DRAM interposer were successfully removed. Remaining is the metallic and silicon based material of the circuitry, connections, and memory with clean surfaces remaining such that the design file could be validated.

**Table 2:**
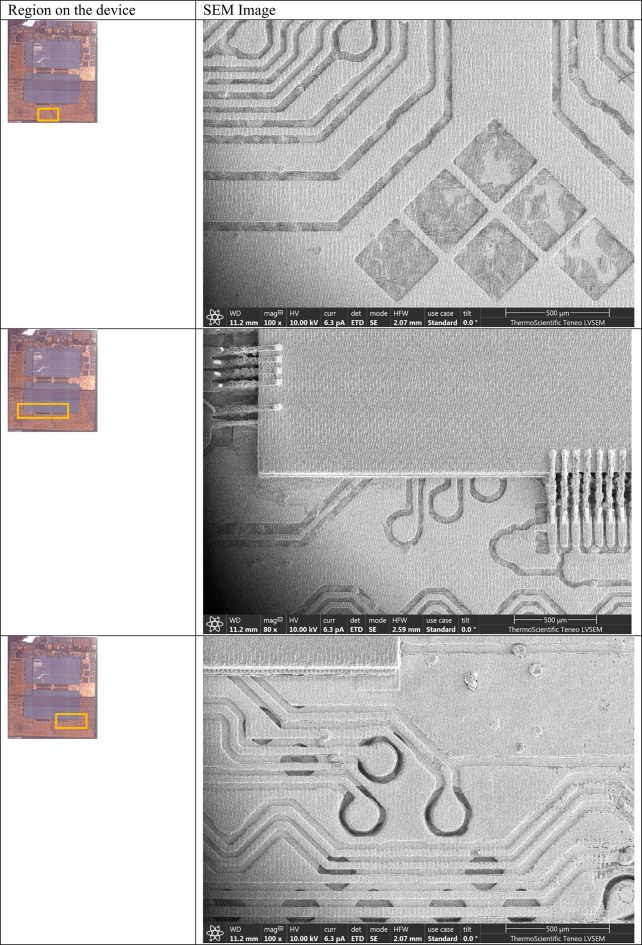

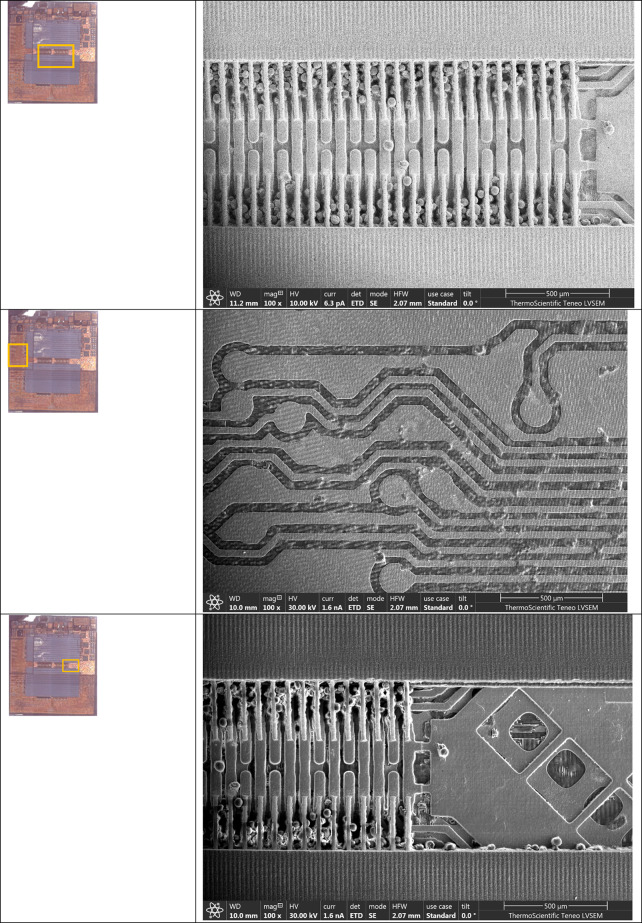

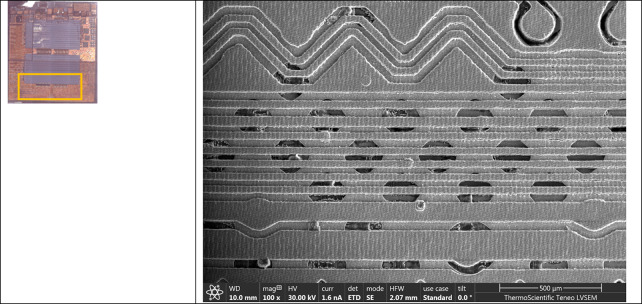
SEM images from different regions of the decapsulated device.

### DRAM removal

**Fig. 6:**
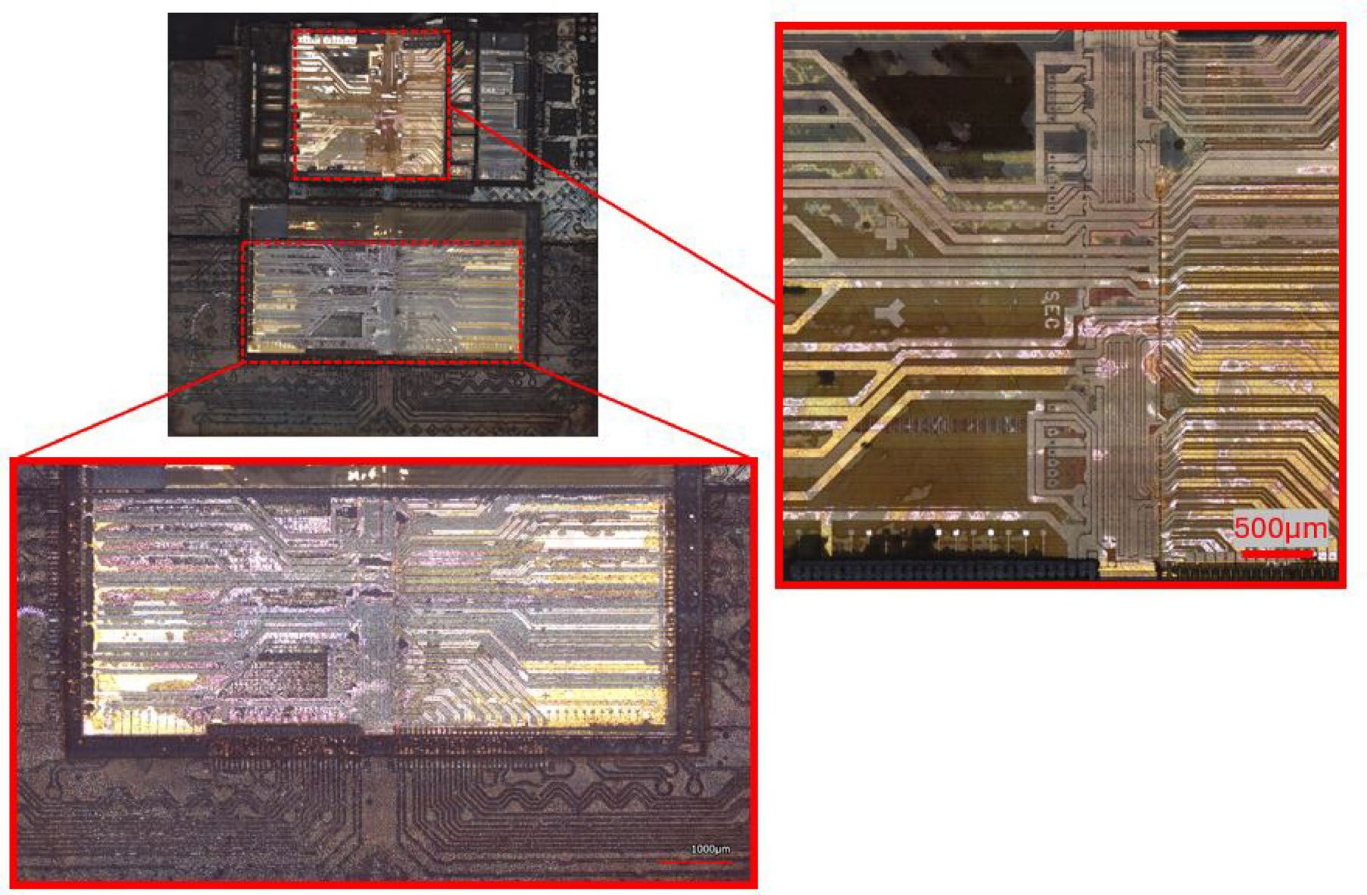
Optical microscopy view of the advanced packaged device after femtosecond-laser removal of the two central rectangular DRAM die components. The exposed regions reveal the underlying package surface, enabling subsequent delayering and high-resolution inspection.

Following decapsulation, the DRAM regions (the two rectangular structures located near the center of the package) were removed using the femtosecond laser delayering process to expose the underlying package layers for subsequent analysis. Figure 6 shows the device surface after DRAM removal using optical imaging. Prior to applying the processes across the entire DRAM, another round of parameter optimization was performed in an array of small rectangular and square regions visible in the lower magnification image. The best parameters were selected from these experiments and applied to the rest of the DRAM region. It was found that a power ~ 40 times higher was required for these materials compared to the molding compound and a reduced pulse spacing (3 ps) performed better.

**Fig. 7:**
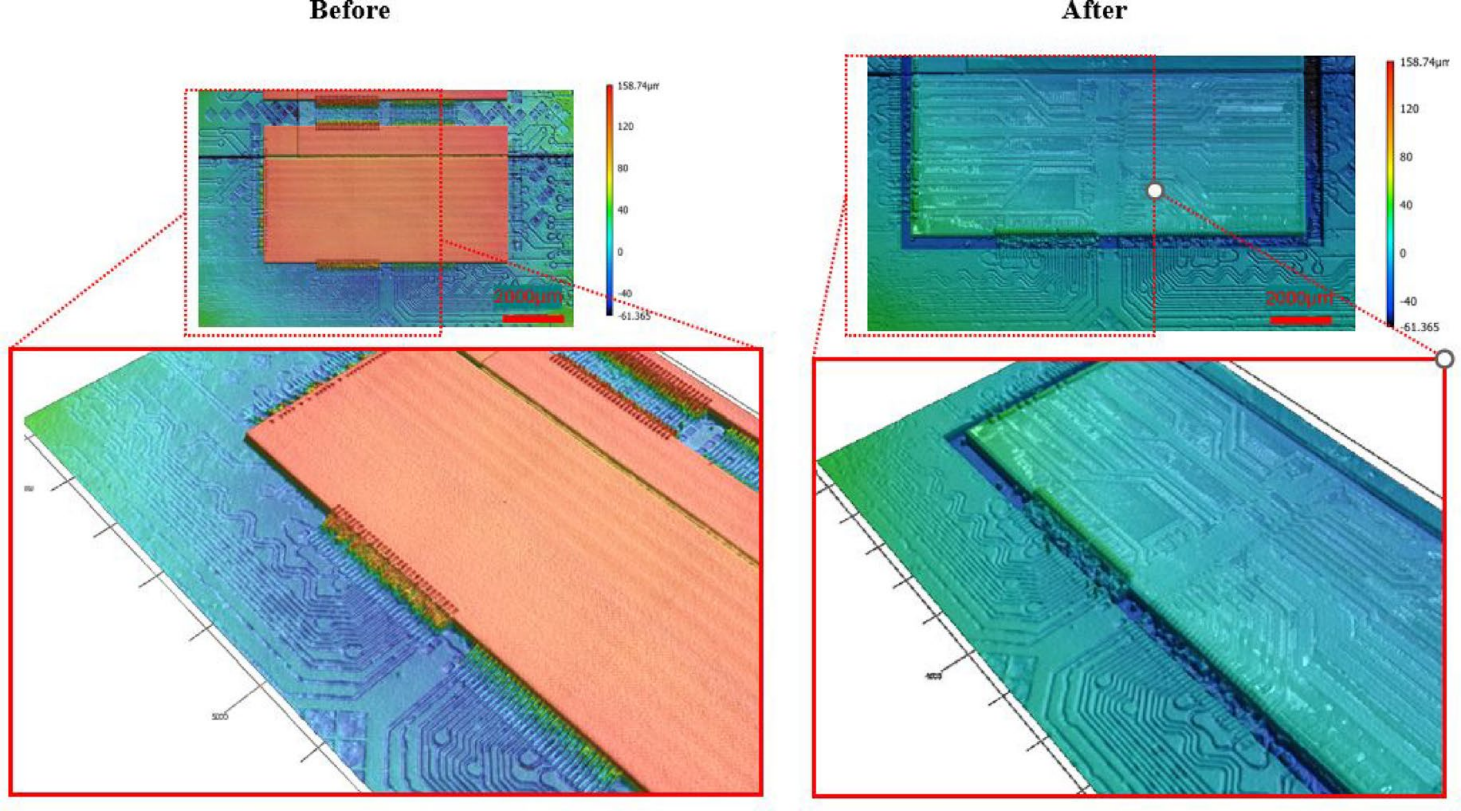
Laser-scanning confocal microscopy comparison before and after DRAM removal. Left: Device surface after decapsulation with DRAM components intact. Right: Same region following femtosecond-laser delayering and DRAM removal, showing clean exposure of the underlying materials. Quantitative surface measurements are provided in Surface Topography Measurement Section.

Figure 7 presents confocal microscopy images comparing the surface morphology before and after removal of the two DRAM dies. Height measurements demonstrate that the area previously under the DRAM is coplanar with the surrounding copper layers. SEM imaging was performed across multiple regions of the exposed surface to evaluate removal quality and confirm clean exposure of underlying features (Table 3).

**Table 3:**
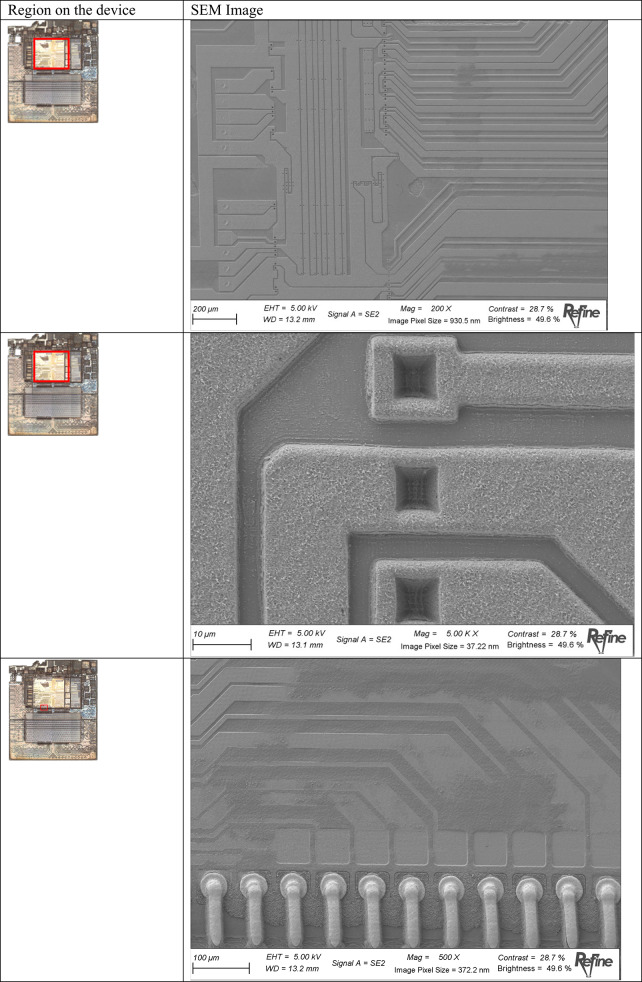
SEM characterization of multiple regions across the package after DRAM removal by femtosecond-laser delayering. Images highlight surface quality and consistency of material removal, demonstrating clean exposure of underlying structures with minimal damage and low residual contamination.

### Interposer/substrate RDL copper layer removal

After DRAM removal, the exposed surface revealed interposer/substrate copper redistribution layers (RDL), characterized by large-area copper routing traces and via/pad arrays. Following removal of the DRAM and the underlying silicon layer, three stacked copper layers became visible and were removed sequentially, one layer at a time, to expose the underlying package features. As with the other materials, tailored parameters were required for the primarily copper layers. Through the same procedure as discussed in the other sections it was determined that longer pulse widths (~ 1 ps) improved metal ablation efficiency, and high-burst modes were not required in this step.

**Fig. 8:**
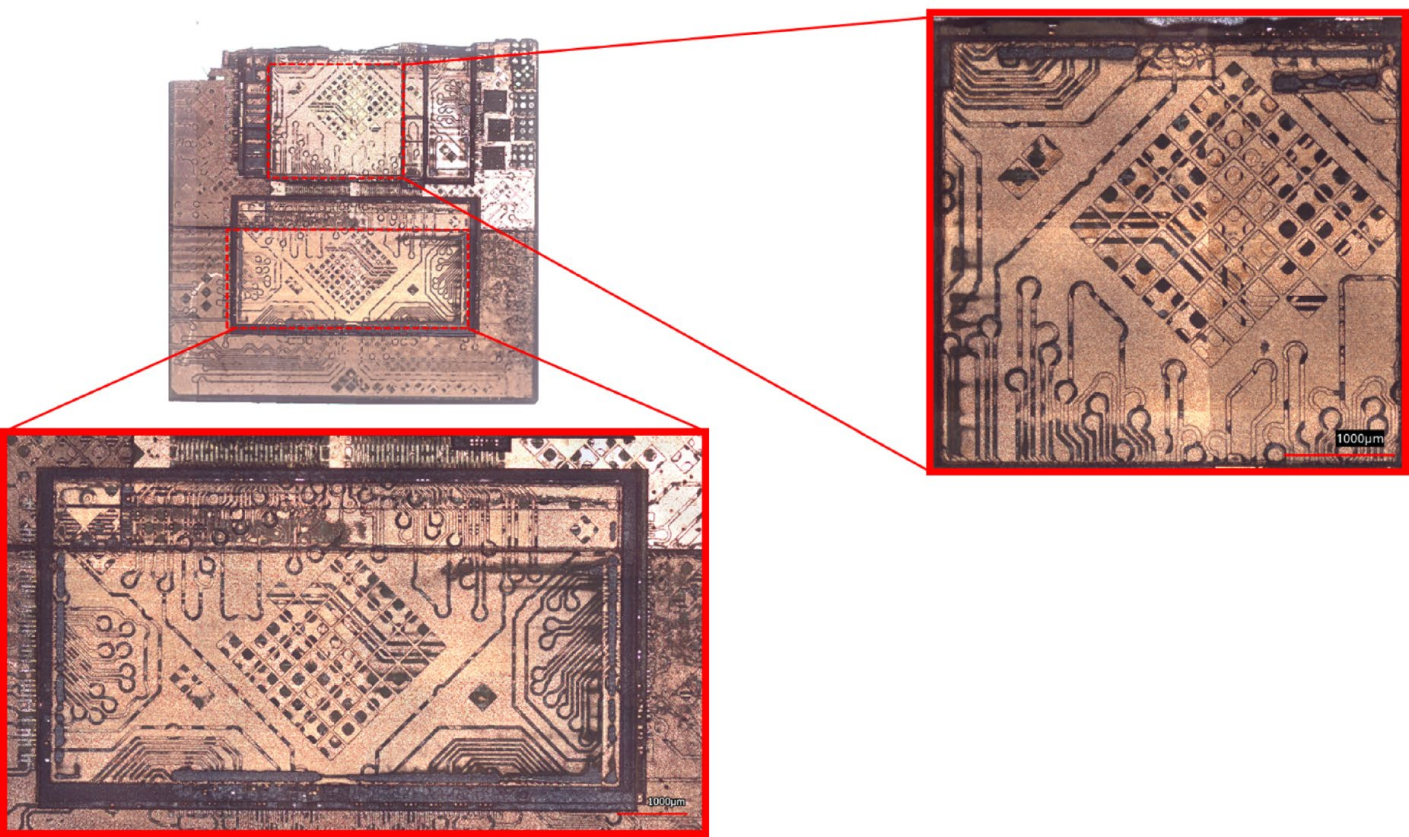
First copper layer imaged optically.

Figure 8 shows optical images of the first copper layer revealed.

### First copper layer

**Fig. 9:**
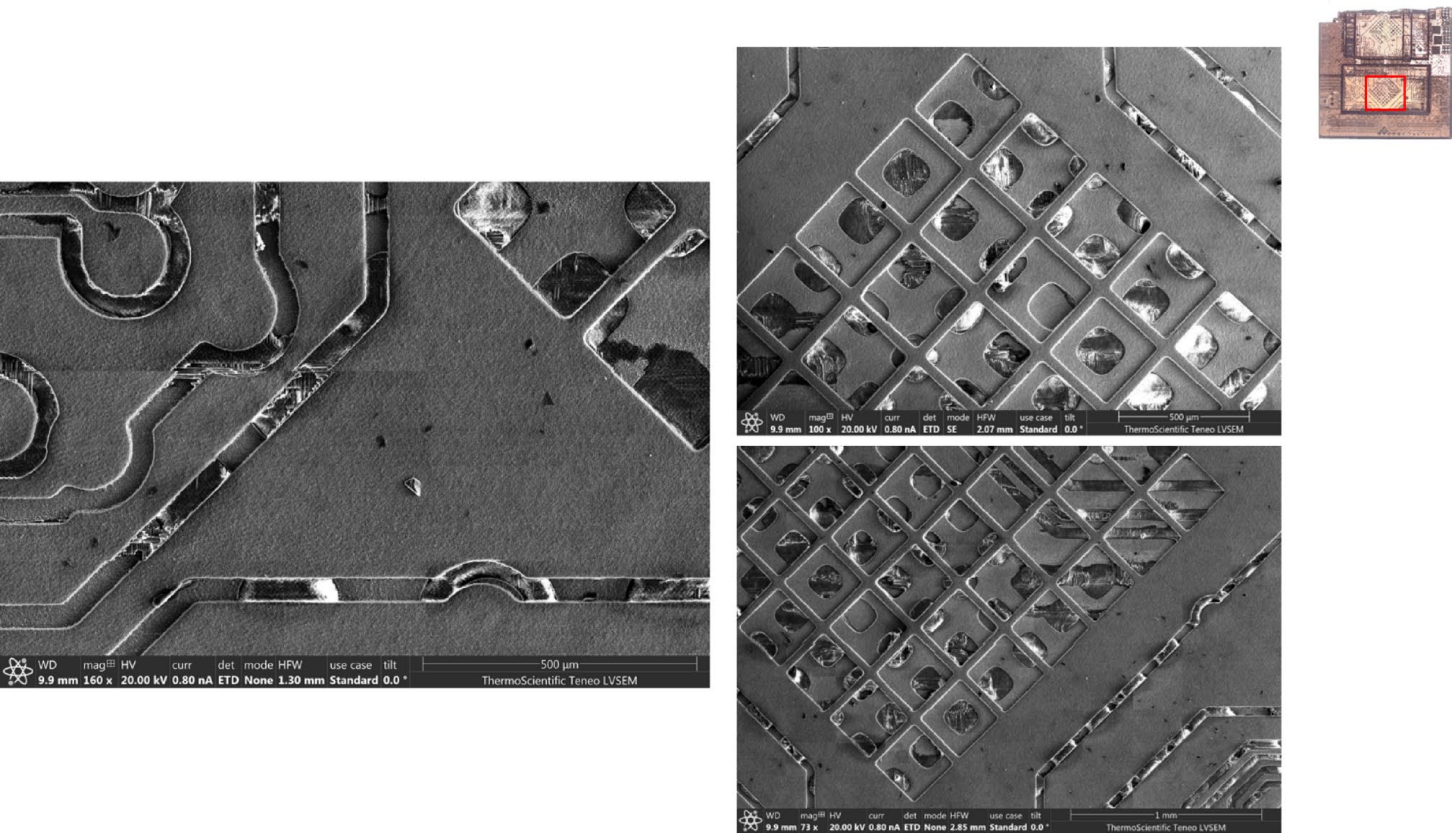
SEM images of the first copper layer revealed.

Figure 9: shows SEM images of the first copper layer.

### Second copper layer

**Fig. 10:**
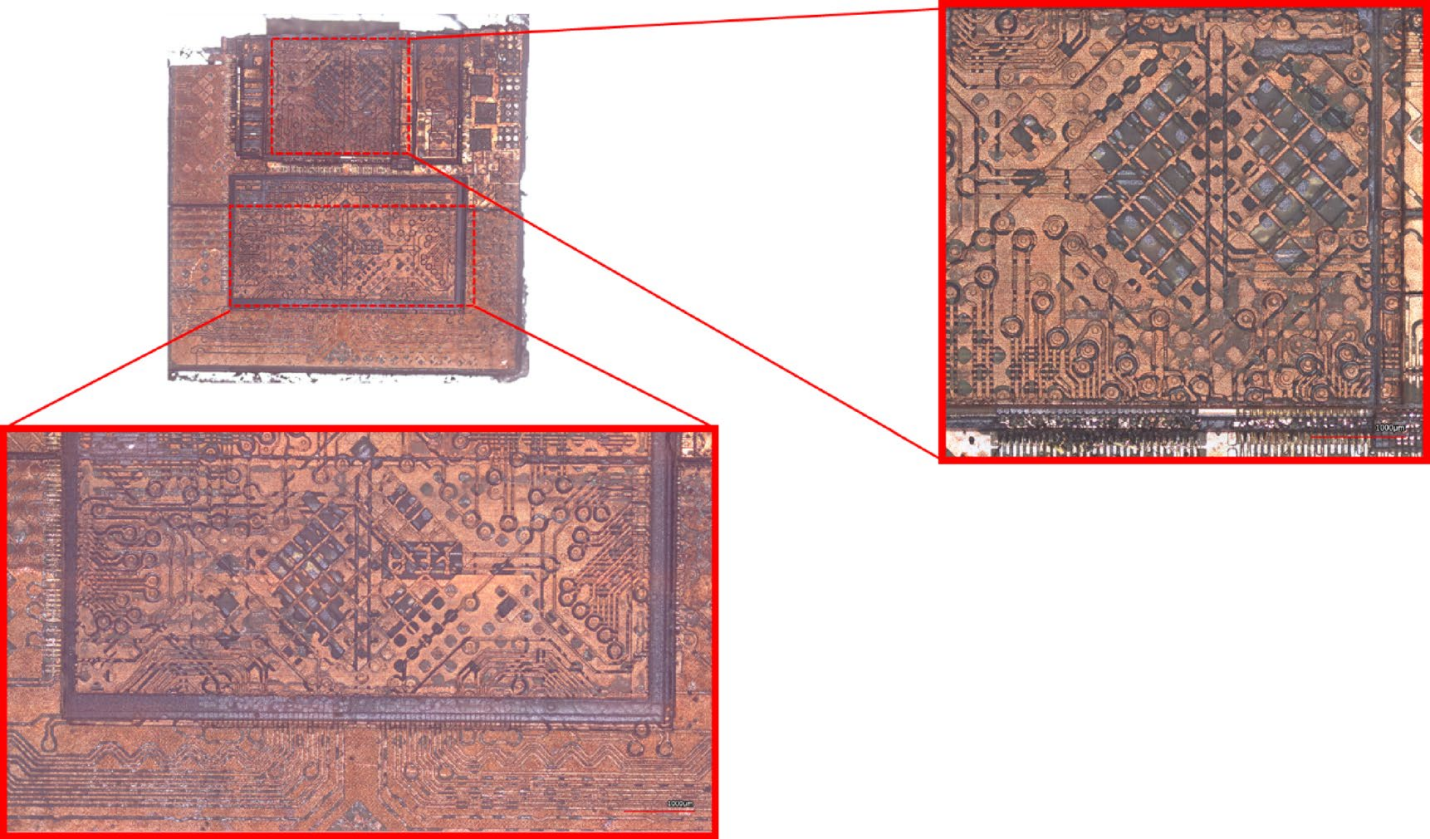
Second copper layer imaged optically.

Figure 10 shows optical images of the second copper layer revealed.

**Fig. 11:**
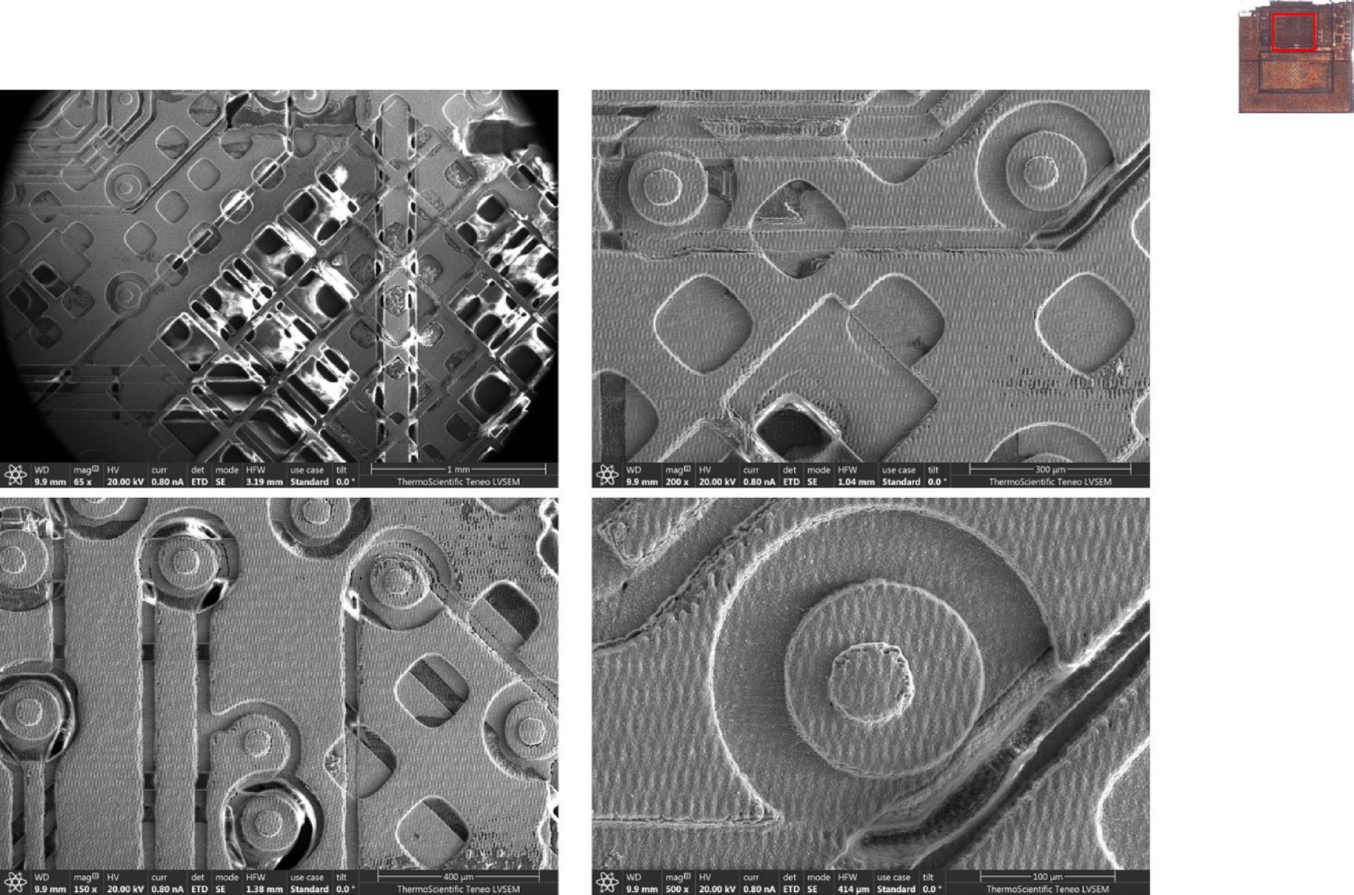
SEM images of the second copper layer revealed.

Figure 11: shows SEM images of the second copper layer.

### Third copper layer

**Fig. 12:**
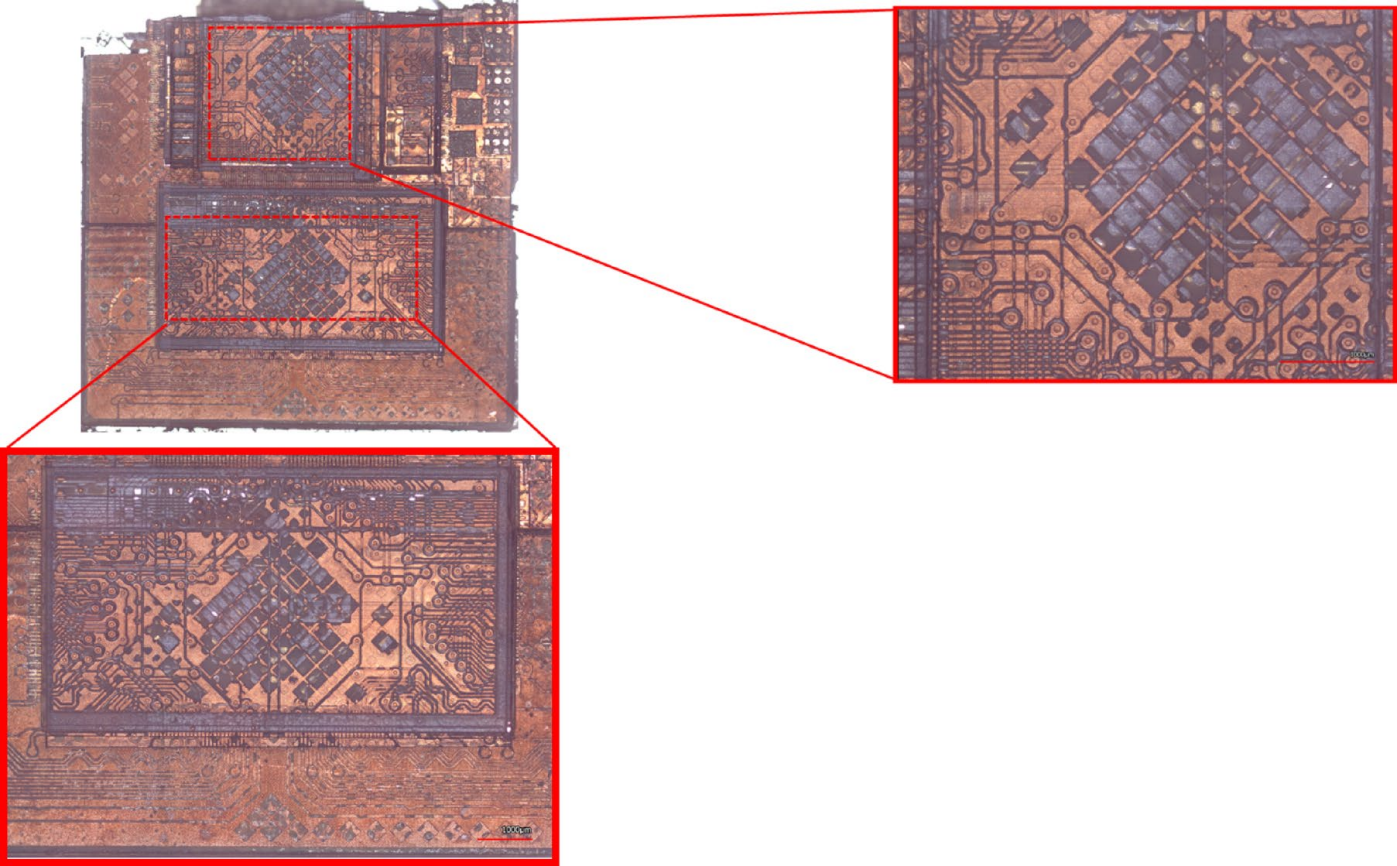
Third copper layer imaged optically.

Figure 12 shows optical images of the third copper layer revealed.

**Fig. 13:**
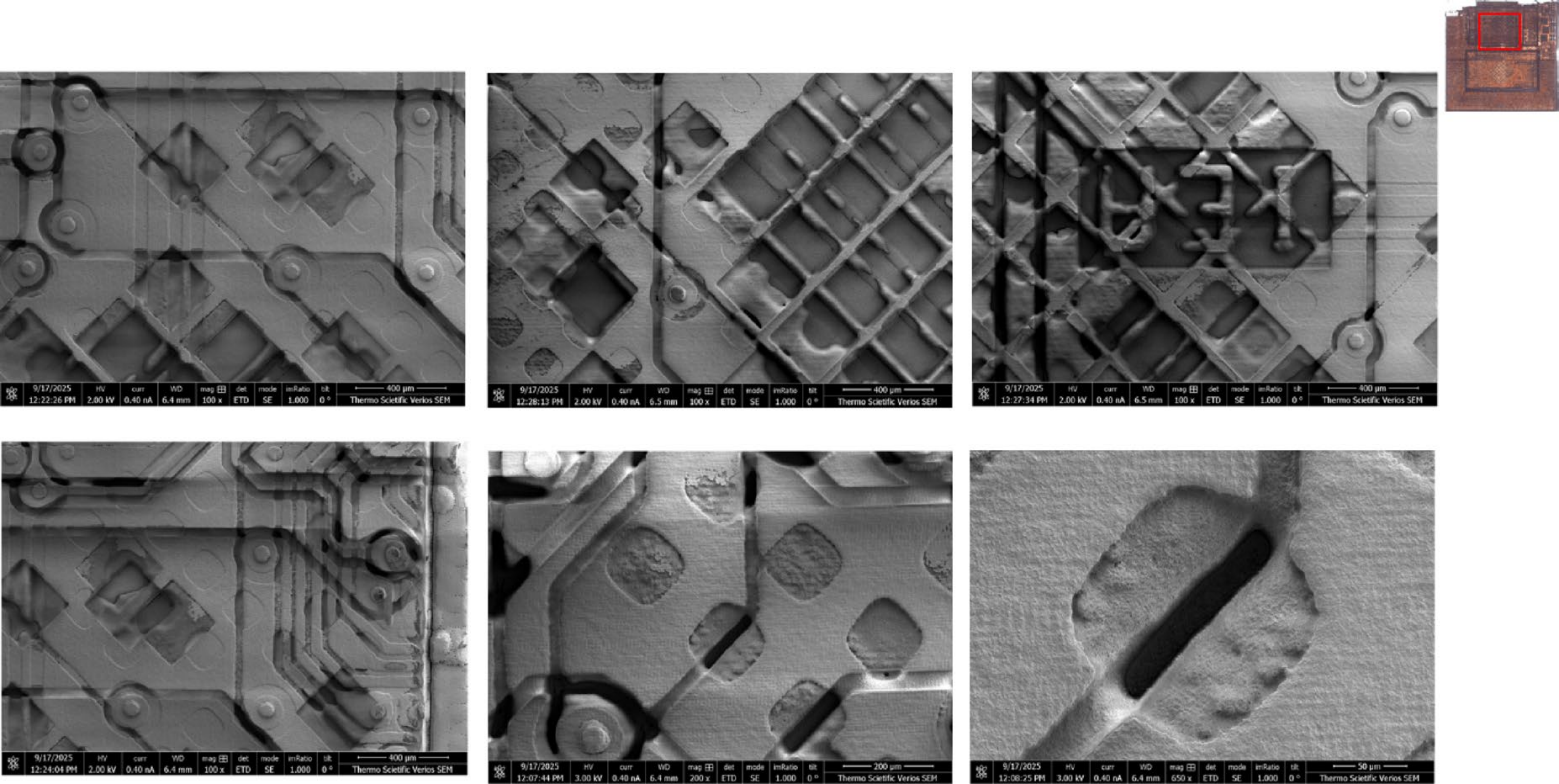
SEM images of the third copper layer revealed.

Figure 13: Shows SEM images of the third copper layer.

### Laser-enabled ic cross-sectioning

**Fig. 14:**
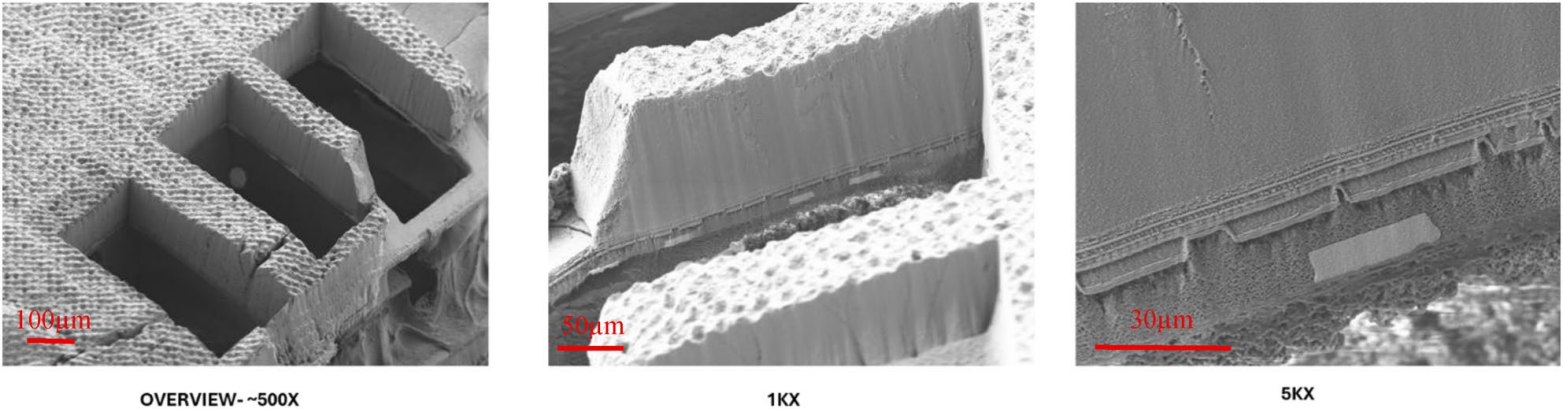
Trenches were cut by laser to create a cross-sectional view of the die.

After sequentially delayering the package layers and reaching the logic die, laser-enabled cross-sectioning was performed to expose the internal device stack for layer-level inspection. This approach enables rapid access to the die cross section and provides a practical alternative to conventional sectioning methods for revealing buried structures and interfaces. Figure 14 shows an SEM image of the femtosecond-laser cross section, illustrating the exposed IC layer architecture and confirming the ability of the process to generate a well-defined section suitable for follow-up analysis.

### Material removal rates

The material-removal rates varied significantly across the package because the epoxy molding compound, DRAM regions, and Cu redistribution layers have different optical, thermal, and ablation responses. These removal rates are as follows: epoxy molding compound (19.2 mm^3^/min); DRAM (0.96 mm^3^/min); Cu-rich RDL layers (0.54 mm^3^/min).

### Surface topography measurements

Confocal roughness measurements were performed on representative material-dominated regions after laser processing. Roughness was not used as the sole quality metric for regions containing exposed circuitry because the measured Sa and Sq can be dominated by real circuit topography, metal traces, vias, or redistribution features rather than process-induced roughness. Therefore, roughness values are reported for representative molding-compound, silicon-rich, and copper-rich regions, while patterned circuitry regions are evaluated using a combination of confocal topography, SEM feature visibility, residual-debris assessment, and layer-exposure quality. The surface measurements values for the representative regions were as follows: silicon (Sa = 3.2 μm); DRAM (Sa = 9.2 μm); Cu (Sa = 12.2 μm).

### Parameter tuning and reproducibility framework

Because the ablation response depends on the full optical and mechanical configuration of the laser-processing system, the optimized numerical parameters should not be interpreted as universally transferable values. The effective fluence and ablation behavior depend on the laser source, pulse compressor settings, beam delivery optics, scanner, F-theta lens, spot size, focus position, assist-gas configuration, cleaning protocol, and sample mounting. Therefore, reproducibility is best supported by reporting the tuning procedure used to converge on the final process conditions.

Parameter tuning was performed on small test regions before full-area processing. For each material class, a matrix of test windows was produced by varying average power/pulse energy, repetition rate, pulse width, scan speed, hatch spacing, number of cycles, burst condition, and intra-burst timing. The resulting test regions were evaluated using optical microscopy, confocal topography, and SEM. The selection metrics included: completeness of layer removal, visibility of exposed features, residual debris, surface continuity, preservation of underlying structures, absence of excessive melting or edge rounding, and processing time.

To support transferability, parameters are reported as approximate ranges and, where appropriate, relative to a baseline recipe. For example, DRAM removal required approximately 8× higher average power than the molding-compound baseline, while molding-compound removal benefited from a 3× higher burst count operation. Copper-rich redistribution layers required a separate metal-optimized condition, where shorter effective pulse interaction with low-burst operation improved metal removal and high-burst operation was not required. See Table 4 for details on the observed qualitative process trends.

**Table 4:** Observed qualitative process trends.

Parameter	Effect of increasing parameter	Practical implication
Average power / pulse energy	Increases removal depth and ablation rate; excessive values may cause roughness, redeposition, or damage	Adjusted according to material type and target selectivity
Repetition rate	Can improve throughput but may increase thermal accumulation	Balanced to maintain both efficiency and surface quality
Scan speed	Higher speed lowers deposited energy per area; lower speed increases interaction	Used to control removal uniformity and minimize substrate damage
Number of passes/cycles	Increases total removed depth	Multiple gentle passes improved control in sensitive regions
Pulse width	Influences confinement of energy deposition and metal interaction	Ultrashort pulses enabled localized ablation with limited heat-affected zones
Burst condition	Affects ablation efficiency and energy coupling	Higher burst counts benefited polymer removal, while lower burst conditions were preferred for semiconductor and metal layers
Intra-burst timing	Modifies interaction between pulses and the evolving surface/plasma	Optimized differently for polymeric and functional device layers
Assist-gas / cleaning	Reduces redeposition and debris accumulation	Improved surface cleanliness and imaging quality

### Layer-specific processing observations

#### Epoxy / Molding compound removal

The molding compound was removed using a high-throughput ablation condition optimized for rapid polymer removal. Our observation was that efficient material removal could be achieved with aggressive processing conditions and minimal scanning cycles. High-burst operation improved energy coupling within the polymer matrix and enhanced removal uniformity while maintaining acceptable surface quality.

#### DRAM layer removal

The DRAM region required a significantly more controlled and selective process because of its fine structures and sensitivity to thermal and mechanical damage. Compared to the molding-compound process, the DRAM removal strategy relied on lower effective energy deposition combined with multiple progressive passes to gradually expose the underlying features. Reduced burst conditions improved process stability and minimized excessive heat accumulation, enabling more controlled layer-by-layer delayering.

#### Copper-rich layer removal

Copper-rich redistribution and interconnect layers required a separate metal-optimized ablation condition due to the high reflectivity and thermal conductivity of Cu. In contrast to the polymer-removal strategy, high-burst operation was not beneficial for metallic regions. Instead, lower-burst or single-pulse conditions provided more stable and localized material removal while reducing redeposition effects. The ultrafast pulse regime enabled controlled Cu removal with minimal collateral damage to adjacent structures.

## Discussion

This work demonstrates an integrated, single-platform femtosecond-laser workflow for advanced package-on-package analysis. The workflow combines decapsulation, package/interposer delayering, DRAM removal, sequential Cu redistribution-layer exposure/removal, and targeted logic-die cross-sectioning. This combination of operations on a single platform and on a single advanced packaged device distinguishes the present work from prior reports that demonstrated individual aspects of laser delayering, laser/FIB preparation, or bare-die analysis. Importantly, the demonstrated scope should be interpreted as package-level deprocessing and targeted access to selected logic-die cross sections, rather than complete layer-by-layer reconstruction of the full logic-die stack.

A key enabler of this capability is the systematic parameter-optimization strategy used throughout the workflow. Because advanced packages contain highly heterogeneous materials—including polymers/epoxies, dielectrics, silicon-rich regions, and metals—no single parameter set is suitable for every layer. The results show that achieving high-quality exposure without damaging underlying structures requires deliberate tuning of laser variables such as power, repetition rate, pulse formatting, burst-mode condition, scan strategy, number of passes, and pulse width, tailored to each material class. Importantly, our optimization approach was not based on ad hoc trial-and-error, but on structured testing over small regions, followed by evaluation using optical microscopy, confocal topography, and SEM. Final conditions were selected based on layer-exposure quality, residual debris, preservation of underlying features, surface continuity, and processing time. This systematic methodology improves reproducibility and supports practical deployment for real FA scenarios where consistency and turnaround time are critical.

Equally important is the integration of multimodality microscopy to validate delayering outcomes and guide subsequent steps. Optical imaging provides rapid feedback over large areas, confocal microscopy enables quantitative topography and fine feature visualization after each delayering step, and SEM offers high-resolution confirmation of interface quality and structural integrity where needed. This combined imaging strategy is essential for ensuring that each laser step achieves the desired exposure state and for preventing error accumulation during multi-stage delayering workflows. In addition, quantitative metrics such as representative material roughness, removal rate, and height variation provide a more objective basis for evaluating process quality. For patterned circuitry regions, these metrics must be interpreted carefully because measured roughness may include true device topography rather than laser-induced surface roughness.

Looking forward, an important next step is extending the demonstrated approach from package-level deprocessing and targeted logic-die cross-sectioning to full die delayering. Such an extension would enable comprehensive layer-by-layer reconstruction through the entire IC stack, including systematic comparison of internal structures across metallization and dielectric layers. The present work therefore establishes a practical workflow for rapid package access, redistribution-layer exposure, and selected die-level inspection, while full logic-die reconstruction remains an important direction for future development.

## Conclusion

In this work, we demonstrated an all-in-one femtosecond-laser workflow for advanced packaged semiconductor failure analysis and structural investigation using an advanced package-on-package device as a showcase. Starting from decapsulation, the process enabled controlled removal of epoxy molding, DRAM components, and interposer/substrate copper redistribution structures, followed by laser-enabled cross-sectioning of the logic die to reveal the internal IC layer architecture. The results confirm that femtosecond-laser processing can provide a rapid, scalable, and effective alternative to conventional delayering approaches for advanced packaging devices, while maintaining clean exposure quality suitable for high-resolution characterization.

A central outcome of this study is that robust performance across heterogeneous package materials is achievable through systematic parameter optimization tailored to each material class (polymers, dielectrics, and metals). When combined with multimodality microscopy—including optical imaging, confocal topography, and SEM validation—the proposed approach forms a practical end-to-end platform for targeted access, delayering, and cross-sectional inspection across multiple length scales. Future efforts will focus on extending this workflow to complete layer-by-layer delayering through the full die stack, enabling comprehensive design reconstruction and deeper failure localization for next-generation advanced packaged systems.

## Data Availability

All data generated or analyzed during this study are included in this published article.
